# Structured Reporting Using CEUS LI-RADS for the Diagnosis of Hepatocellular Carcinoma (HCC)—Impact and Advantages on Report Integrity, Quality and Interdisciplinary Communication

**DOI:** 10.3390/cancers13030534

**Published:** 2021-01-31

**Authors:** Thomas Geyer, Johannes Rübenthaler, Constantin Marschner, Malte von Hake, Matthias P. Fabritius, Matthias F. Froelich, Thomas Huber, Dominik Nörenberg, Johannes Rückel, Maximilian Weniger, Corinna Martens, Laura Sabel, Dirk-André Clevert, Vincent Schwarze

**Affiliations:** 1 Department of Radiology, University Hospital, LMU Munich, 81377 Munich, Germany; johannes.ruebenthaler@med.uni-muenchen.de (J.R.); constantin.marschner@med.uni-muenchen.de (C.M.); maltevonhake@gmail.com (M.v.H.); matthias.fabritius@med.uni-muenchen.de (M.P.F.); johannes.rueckel@med.uni-muenchen.de (J.R.); lauranina.sabel@gmail.com (L.S.); dirk.clevert@med.uni-muenchen.de (D.-A.C.); vincent.schwarze@med.uni-muenchen.de (V.S.); 2 Department of Radiology and Nuclear Medicine, University Medical Center Mannheim, 68167 Mannheim, Germany; matthias.froelich@medma.uni-heidelberg.de (M.F.F.); thomas.huber@medma.uni-heidelberg.de (T.H.); dominik.noerenberg@medma.uni-heidelberg.de (D.N.); 3 Department of General, Visceral, and Transplantation Surgery, University Hospital, LMU Munich, 81377 Munich, Germany; maximilian.weniger@med.uni-muenchen.de; 4 Department of Medicine II, University Hospital, LMU Munich, 81377 Munich, Germany; Dr.C.Martens@mvzimkloster.de

**Keywords:** structured reporting, hepatocellular carcinoma, CEUS, clinical decision-making, contrast-enhanced ultrasound

## Abstract

**Simple Summary:**

Contrast-enhanced ultrasound (CEUS) is an increasingly accepted imaging modality for visualizing hepatocellular carcinoma (HCC) and is recommended as a secondary imaging option by most leading hepatology societies. In recent years, the use of structured reporting (SR) has been recommended by several societies to standardize report content and improve report quality of various diagnostic modalities when compared to conventional free-text reports (FTR). Our single-center study aimed to evaluate the use of SR using a CEUS LI-RADS software template in CEUS examinations of 50 HCC patients. SR significantly increased report integrity, satisfaction of the referring physicians, linguistic quality and overall report quality compared to FTR. Therefore, the use of SR in CEUS examinations of HCC patients may represent a valuable tool to facilitate clinical decision-making and improve interdisciplinary communication in the future.

**Abstract:**

Background: Our retrospective single-center study aims to evaluate the impact of structured reporting (SR) using a CEUS LI-RADS template on report quality compared to conventional free-text reporting (FTR) in contrast-enhanced ultrasound (CEUS) for the diagnosis of hepatocellular carcinoma (HCC). Methods: We included 50 patients who underwent CEUS for HCC staging. FTR created after these examinations were compared to SR retrospectively generated by using template-based online software with clickable decision trees. The reports were evaluated regarding report completeness, information extraction, linguistic quality and overall report quality by two readers specialized in internal medicine and visceral surgery. Results: SR significantly increased report completeness with at least one key feature missing in 31% of FTR vs. 2% of SR (*p* < 0.001). Information extraction was considered easy in 98% of SR vs. 86% of FTR (*p* = 0.004). The trust of referring physicians in the report was significantly increased by SR with a mean of 5.68 for SR vs. 4.96 for FTR (*p* < 0.001). SR received significantly higher ratings regarding linguistic quality (5.79 for SR vs. 4.83 for FTR (*p* < 0.001)) and overall report quality (5.75 for SR vs. 5.01 for FTR (*p* < 0.001)). Conclusions: Using SR instead of conventional FTR increases the overall quality of reports in CEUS examinations of HCC patients and may represent a valuable tool to facilitate clinical decision-making and improve interdisciplinary communication in the future.

## 1. Introduction

Among malignant primary liver tumors, hepatocellular carcinoma (HCC) is the most common type with more than 80% of liver cancers being HCC [1]. Unlike many other solid malignant tumors, HCC can be diagnosed by imaging only without additional tissue sampling needed. In contrast-enhanced imaging, HCC lesions predominantly are characterized by a typical enhancing pattern with early arterial enhancement and venous wash-out which can be detected by computed tomography (CT), magnetic resonance imaging (MRI) or contrast-enhanced ultrasound (CEUS) [2,3]. Using CEUS to detect this typical enhancing pattern and diagnose HCC brings several benefits such as high diagnostic accuracy, high safety, fast availability and cost-effectiveness [4,5,6]. Due to its benefits and its high diagnostic accuracy, CEUS is increasingly accepted as an imaging modality for visualizing HCC, hence it is recommended as a secondary imaging option by most leading hepatology societies [3,7,8,9]. High accuracy and integrity of CEUS reports are therefore pivotal for patient management and guiding adequate treatment.

Recently, the use of structured reporting (SR) has been recommended by several medical societies to standardize report content and improve report quality of various diagnostic modalities when compared to conventional free-text reports (FTR) and thereby simplify clinical decision-making [10,11,12,13]. Various studies on different medical imaging modalities have shown that SR can facilitate clinical decision-making by improving the quality, accuracy and integrity of radiology reports, and reduce reporting times. Therefore, both radiologists and referring physicians favored SR over FTR in these studies [14,15,16,17,18]. Especially when used by inexperienced residents, SR may lead to more thorough and comprehensive reports [19,20,21]. Additionally, a previous study demonstrated that by using SR for head and neck ultrasound examinations, the process of acquiring ultrasound skills in medical education was accelerated [22]. Furthermore, previous studies indicated that SR may facilitate the use of artificial intelligence algorithms and might therefore be beneficial for scientific data analyses [14,23].

While several studies in the past examined the effects of using SR in different imaging modalities, no studies have been conducted so far to investigate the value of SR in CEUS. The present study aimed to evaluate the influence of SR in CEUS examinations of HCC patients on report quality, comprehensibility, completeness and overall satisfaction of the referring physicians to improve patient management and interdisciplinary communication in the future.

## 2. Materials and Methods

### 2.1. Study Design

**Figure 1:**
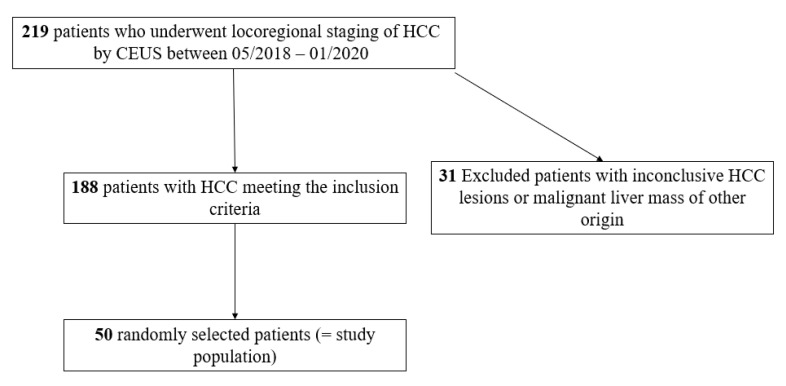
Flowchart illustrating included and excluded patients who underwent contrast-enhanced ultrasound (CEUS) for locoregional hepatocellular carcinoma (HCC) assessment.

Our retrospective single-center study was approved by the institutional review board. All authors declared that they followed the ethical guidelines for publication in Cancers. All data used for this study were collected according to the principles of the Declaration of Helsinki/Edinburgh 2002. We retrospectively searched our institutional database of radiology reports to identify HCC patients who underwent CEUS examinations for assessing the locoregional tumor status of the liver at our University Hospital. We included 50 randomly selected patients who underwent CEUS (Figure 1). Only patients with precise information about the referring physician‘s clinical questions were included.

### 2.2. Sample Size Calculation

We calculated the required number of reports based on anticipated effect size when comparing the rate of SR and FTR with completeness of 80% or higher. We estimated that 55% of FTR would receive high or very high (>80%) completeness ratings based on previous publications comparing SR to FTR in radiology [21,22,24]. Furthermore, we estimated that SR would increase the number of high or very high ratings up to 70%. Based on these assumptions, the required effect size was *n* = 82 (41 SR, 41 FTR) with a power of 80% and a level of significance of *p* = 0.05. Accounting for a possible overestimation of the effect size, we increased our sample size to *n* = 100 (50 SR, 50 FTR).

### 2.3. Image Acquisition

**Figure 2:**
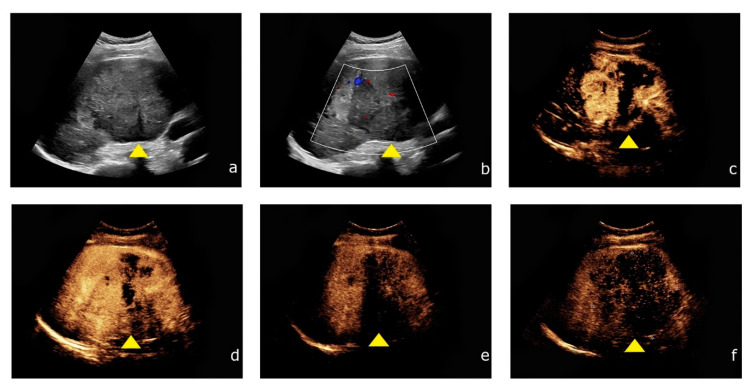
Contrast-enhanced ultrasound of HCC showing the typical contrast enhancement pattern. (**a**) Inhomogeneous liver lesion (yellow arrowhead) in the right liver lobe (8.7 cm) in native B-mode. (**b**) Intralesional hypervascularization is detected by Color Doppler. (**c**,**d**) After intravenous application of SonoVue^®^ (Bracco, Milan, Italy) the lesion shows rapid contrast enhancement during the arterial phase. (**e**,**f**) During the venous phase, the lesion shows typical wash-out during the venous phase.

All CEUS examinations used for this study were performed by one experienced consultant radiologist (EFSUMB Level 3). All examinations contained B-mode, Color Doppler, and CEUS following specific CEUS protocols, and were performed using up-to-date high-end ultrasound systems (Samsung RS80 and RS 85, Samsung Medison, Seoul, Korea Philips EPIQ 7, Seattle, WA, USA) and a low mechanical index (<0.2) to avert early destruction of microbubbles. For all CEUS examinations, we used the second-generation blood pool contrast agent SonoVue® (Bracco, Milan, Italy). One point six to two point four milliliters of SonoVue were applied intravenously followed by a bolus of a 5–10 mL sterile 0.9% sodium chloride solution. Liver lesions were assessed during the early arterial phase (10–45 s after application of SonoVue), the portal venous phase (45–120 s) and the late venous phase (2–6 min) (Figure 2). Image quality was sufficient in all examinations. Acquired data were archived as cine-loops in our picture archiving and communication system (PACS).

### 2.4. FTR and SR

All FTR used for our study were created in clinical routine by the consultant radiologist who performed CEUS examinations. Reports were created by using a speech recognition software system without using structured templates or predefined text modules (Philips SpeechMagic 6.1, Build 543 SP1 (7/2007), Philips Speech Processing Solutions, Vienna, Austria).

**Figure 3:**
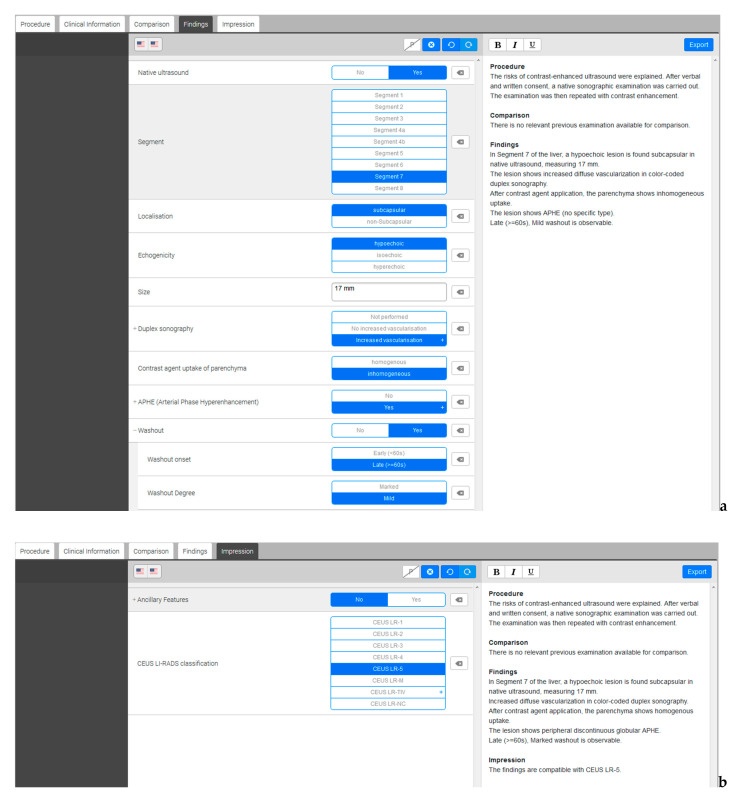
Screenshots of the structured reporting template. (**a**) On the left side, the software offers decision trees with several clickable items and subitems. On the right side, full semantic sentences are generated automatically. (**b**) In a further step, the assessed liver lesion can be categorized according to the CEUS LI-RADS classification on the left side. On the right side, the selected category appears at the end of the generated report.

For creating SR, we built a structured reporting template by using online software for structured reporting (Smart Reporting GmbH, Munich, Germany, http://www.smart-reporting.com). This structured template contains clickable decision trees which we created based on the CEUS Liver Imaging Reporting and Data System (CEUS LI-RADS, illustrated in Table 1) by the American College of Radiology (ACR) [25]. By clicking on adequate items in the decision tree, the software automatically generates semantic sentences from predefined text blocks (Figure 3). The semantic sentences can be exported from the template for the SR without further need for manual adjustment. However, additional information can be added manually by the examiner if necessary. All SR were generated retrospectively by experienced radiologists from our hospital (five years of professional experience) after scrutinizing the archived cine-loops from CEUS and analyzing the patient information and relevant questions provided by the referring clinicians.

### 2.5. Report Evaluation

**Figure 4:**
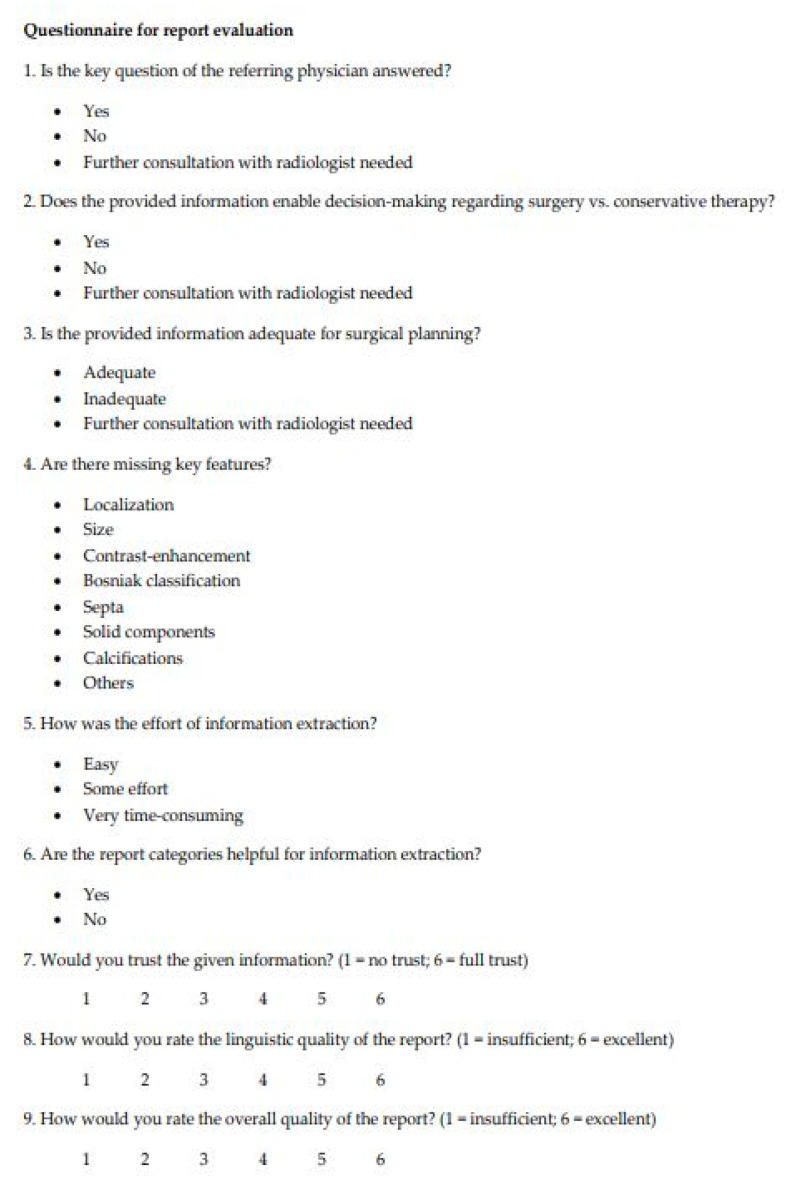
Questionnaire used for the evaluation of structured reports and free-text reports by the two reviewers.

For evaluating report quality and its impact on clinical decision making, we created a questionnaire (Figure 4). The questions analyzed if the referring physician’s key question was answered by the report, the report’s impact on clinical decision making, its completeness and the effort for information extraction. Furthermore, a 6-point Likert scale (1 = insufficient, 6 = excellent) was used to assess the referring physician’s trust in the reports, linguistic quality and overall report quality. One hundred reports (50 FTR, 50 SR) were randomized and anonymized. The questionnaires were filled out by two referring physicians with over seven years of professional experience, one from the institutional Department of Internal Medicine and one from the institutional Department of Visceral Surgery, immediately after reading the reports. Both reviewers were unbiased and evaluated the reports independently.

### 2.6. Statistical Analysis

A McNemar test was used to compare binomial data and a Wilcoxon signed-rank test was used to test the answers on the Likert scales for significance. The level of significance was set at *p* = 0.05. All statistical calculations were performed using IBM SPSS Statistics Version 25 (IBM, Armonk, NY, USA).

## 3. Results

We included 50 patients (37 men and 13 women) with suspected HCC in our study who underwent CEUS between 05/2018 and 01/2020 with a mean age of 63 years at the time of the examination (range: 20–83 years). The mean size of the detected HCC lesions was 3.0 cm (range: 0.7–8.0 cm). Both reviewers filled in 50 questionnaires on SR and 50 questionnaires on FTR (100 SR and 100 FTR cases in total). All 200 questionnaires were filled in completely by the two reviewers.

Key questions of the referring physicians were answered in 100% of SR vs. 95% of FTR (*p* = 0.063). The given information was sufficient for decision-making regarding surgery vs. conservative therapy in 98% of SR vs. 96% or FTR (*p* = 0.688) and information was adequate for surgical planning in 98% of SR vs. 94% of FTR (*p* = 0.289).

Using SR significantly increased report completeness with at least one key feature missing in 31% of FTR vs. 2% of SR (*p* < 0.001). Information extraction was considered easy in 98% of SR vs. 86% of FTR (*p* = 0.004). Among SR, information extraction was related to some effort in 2% of the cases, whereas some effort was necessary for 9% of FTR. In 5% of FTR, information extraction was even considered time-consuming, in contrast to no SR cases (*p* = 0.063). The report structure was helpful in 100% of SR vs. 91% of FTR (*p* = 0.004).

**Figure 5:**
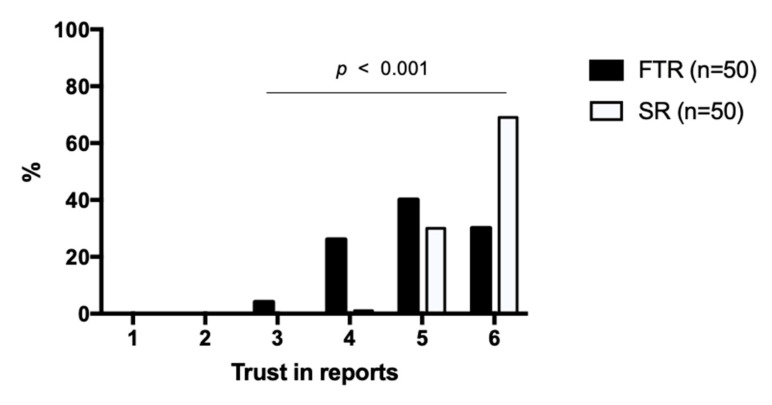
Trust of referring physicians in the report. Structured reports and free-text reports were rated based on a Likert scale ranging from 1 to 6 (1 = insufficient, 6 = excellent). The diagram illustrates the degree of the referring physician’s trust on the x-axis and the percentage distribution on the y-axis. SR = structured reports, FTR = free-text reports.

The trust of referring physicians in the report was significantly increased by SR with a mean of 4.96 ± 0.85 (95% confidence interval (CI): 4.79–5.13) for FTR vs. 5.68 ± 0.49 (CI: 5.58–5.78) for SR (*p* < 0.001) (Table 2, Figure 5).

**Figure 6:**
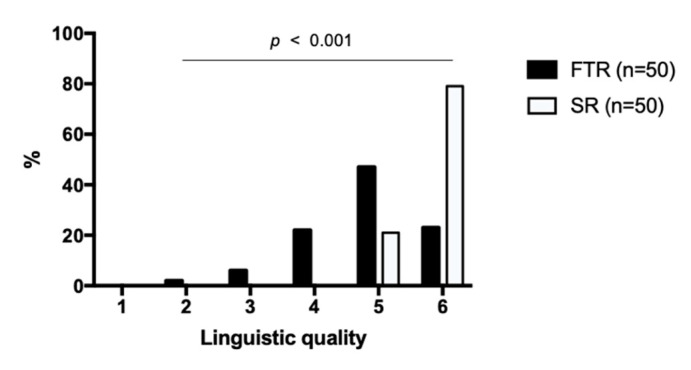
Linguistic quality of the reports. Structured reports and free-text reports were rated based on a Likert scale ranging from 1 to 6 (1 = insufficient, 6 = excellent). The diagram illustrates the degree of the linguistic quality on the x-axis and the percentage distribution on the y-axis. SR = structured reports, FTR = free-text reports.

Linguistic quality of the reports was rated significantly higher in SR 5.79 ± 0.41 (CI: 5.71–5.87) compared to FTR 4.83 ± 0.92 (CI: 4.65–5.01) (*p* < 0.001) (Figure 6). All SR received a rating of 5 (21%) or 6 (79%) on the Likert scale, whereas FTR also received ratings of 4 (22%), 3 (6%) and even 2 (2%).

**Figure 7:**
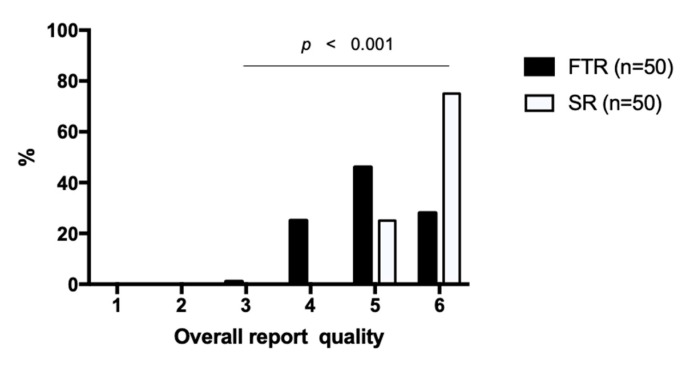
Overall quality of the reports. Structured reports and free-text reports were rated based on a Likert scale ranging from 1 to 6 (1 = insufficient, 6 = excellent). The diagram illustrates the degree of the overall report quality on the x-axis and the percentage distribution on the y-axis. SR = structured reports, FTR = free-text reports.

Overall report quality was rated 5.01 ± 0.76 (CI: 4.86–5.16) for FTR vs. 5.75 ± 0.44 (CI: 5.66–5.84) for SR (*p* < 0.001) (Figure 7). For all SR, overall quality was rated 5 (25%) or 6 (75%) on the Likert scale. The most frequent rating for FTR was 5 (47%) with 28% rated higher (6) but 26% rated lower (3 or 4).

## 4. Discussion

The use of CEUS for diagnosing HCC has increasingly gained widespread acceptance and has been recommended by most leading hepatology societies, as it brings several benefits such as high diagnostic accuracy with high temporal and spatial resolution, fast availability and repeatability, cost-effectiveness and an excellent safety profile. Therefore, high accuracy and integrity of CEUS reports are essential for patient management and adequate clinical decision-making. Recently, several studies have shown that using SR can improve quality, accuracy and integrity of radiology reports compared to FTR in various medical imaging modalities [14,16,18,21,26].

However, no studies have been conducted to compare the use of SR and FTR in CEUS examinations so far. Our study showed that using SR in CEUS examinations of HCC patients significantly increased report completeness, linguistic quality, the trust of referring physicians in the reports and overall report quality compared to conventional FTR. Notably, having only archived cine-loops for preparing SR could have plausibly been a significant disadvantage compared to the radiologist who could guide real ultrasound examinations before creating FTR. As our results clearly demonstrate, SR increased report integrity, satisfaction of the referring physicians, linguistic quality and overall report quality despite this disadvantage. Therefore, the overall quality of SR in locoregional HCC assessment by CEUS may be superior to the quality of FTR. However, these findings need to be further validated in further studies. The overall preference of SR compared to FTR by referring physicians is in line with results from previous publications on various imaging modalities such as computed tomography, magnetic resonance imaging and x-ray [15,16,17,18,26]. One publication on the value of SR compared to FTR in conventional head and neck ultrasound showed similar results [14].

In our study, using SR significantly simplified information extraction, which may be one important factor why clinicians tend to prefer SR over FTR. This supports several previous studies which showed that SR facilitated information extraction for referring physicians [16,21,26]. While clinicians seem to profit from SR, since extracting critical information from radiology reports becomes less time-consuming, it remains unclear if SR also leads to decreased reporting times for radiologists. Some studies reported improved time-efficiency by using SR, whereas other authors described prolonged reporting times for the use of SR compared to FTR [18,22,27,28]. However, it remains to be noted that all of these studies followed a similar pattern and compared conventional narrative reporting to reports created by template-based software with clickable decision trees only. In the future, it might be promising to integrate SR software systems into existing FTR systems to combine the advantages of both reporting approaches. One study evaluating the implementation of such flexible software in a children’s hospital reported high satisfaction rates amongst radiologists [29].

Additionally, we showed that missing key features occurred significantly more often in FTR compared to SR. Since the used software for SR automatically reminds radiologists of every key feature while creating the report, it seems plausible that SR decreases the likelihood of missing one of these features. This might be useful especially for more inexperienced radiologists and medical students who could benefit from a given systematic reporting structure in their learning process. Furthermore, oncological patients could particularly benefit from higher standardization and completeness of radiology reports, as they usually undergo several follow-up examinations. The American College of Radiology introduced the CEUS Liver Imaging Reporting and Data System (CEUS LI-RADS) in 2016 for HCC screening with CEUS, which allows for more standardized terminology and structured interpretation, reduces variability and missed findings in radiology reports and facilitates interdisciplinary communication [25]. Structured reporting might be another important factor that leads to better comparability of radiology reports and therefore facilitates clinical decision-making.

Over the coming years, radiology may face major changes, since machine learning algorithms and radiomics offer a promising approach for increasing efficiency in clinical routine by introducing automated decision support systems and facilitating radiology research [30,31]. Conventional narrative reporting leads to a wide variety of report structures and thus represents an obstacle to the implementation of machine learning software in research [32]. Therefore, standardized reporting may be a valuable tool for facilitating information extraction by automated algorithms and thereby advancing radiology research in the future.

Nevertheless, some authors reported contradictory findings in comparing SR to FTR. A previous study evaluating the use of SR in magnetic resonance imaging of stroke patients showed no significant difference between SR and FTR regarding report accuracy and completeness [27]. However, some contradictory studies have pointed to the possible disadvantages of using SR. Some authors assume that SR could oversimplify radiology reports, decrease diagnostic accuracy and distract radiologists by introducing additional software [10,28,33]. Additionally, radiologists who are used to FTR may experience increased reporting times with SR, especially in the beginning. Thus, SR cannot be considered superior to FTR in general, but must be evaluated for each imaging modality and each structured template separately.

There are several limitations in our study that must be acknowledged. First, SR were not created in clinical practice and not simultaneously with FTR due to the retrospective setting of our study. Second, all FTR were created by one experienced radiologist (European Federation of Societies for Ultrasound in Medicine and Biology Level 3). A variety of different reporting individuals might be helpful to find out if the superiority of SR compared to FTR regarding completeness, comprehensibility and overall report quality can be generalized independent of the individual reporting radiologist. Third, the number of readers, since the reports were evaluated by only two physicians. We aimed to reduce this limitation by separating them in the reviewing process and by not expressing any expectations regarding the potential results of our study. Since the questionnaire evaluated, among other topics, the report’s influence on clinical decision-making regarding conservative vs. surgical therapy, we aimed to maximize clinical expertise by choosing both experts in internal medicine and visceral surgery for reviewing the reports. It also has to be acknowledged that all SR used for this study were created by using a clickable decision-tree. However, there are approaches of SR that are not templated-based as ours but provide a hierarchical reporting structure, e.g., in the form of a checklist and thus include more free-text elements. Hence, our study cannot be generalized to all kinds of SR used by radiologists.

Further prospective studies on using SR in the clinical routine with multiple radiologists creating reports as well as multiple clinicians for report evaluation might provide further evidence on the benefits of SR for CEUS examinations. However, a synchronous structured and unstructured reporting approach in a prospective study setting could be severely biased: given that structured reporting guides through the reporting process, it may also affect the free-text reporting style. Moreover, blinding of reporters or evaluators is not possible. Our retrospective study design goes in line with previous studies investigating SR [14,15,16,21,26]. Yet, we could imagine that the application of SR as an addon—instead of head-to-head FTR vs. SR—in a prospective clinical trial could be more appropriate than a prospective imaging-only study. As reporting styles might need to be adjusted for an individual clinical situation and referring physician, an imaging-only SR-study may not be feasible. 

## 5. Conclusions

In conclusion, the present study shows that using template-based SR instead of conventional FTR increases completeness, comprehensibility and overall quality of radiology reports in CEUS examinations of HCC patients and may thus represent a valuable tool in facilitating clinical decision-making and improve interdisciplinary communication in the future.

## Figures and Tables

**Table 1:** CEUS LI-RADS categorization and diagnostic algorithm according to the American College of Radiology (ACR) [25].

**Categorization**				
CEUS LR-NC	No categorization due to image degradation or omission			
CEUS LR-TIV	Tumor in vein			
CEUS LR-1	Definitely benign			
CEUS LR-2	Probably benign			
CEUS LR-M	Probably or definitely malignant, but not HCC specific			
CEUS LR-3	Intermediate malignancy probability			
CEUS LR-4	Probably HCC			
CEUS LR-5	Definitely HCC			
**Diagnostic Algorithm in CEUS**				
Arterial phase hyperenhancement (APHE)	No APHE		APHE (not rim APHE, not peripheral discontinuous globular APHE)	
Lesion size (mm)	<20	≥20	<10	≥10
No washout	CEUS LR-3	CEUS LR-3	CEUS LR-3	CEUS LR-4
Late and mild washout	CEUS LR-3	CEUS LR-4	CEUS LR-4	CEUS LR-5

**Table 2:** Overview of the Likert scale ratings (1 = insufficient, 6 = excellent) for free-text reports (FTR) and structured reports (SR) regarding trust of the referring physicians in the reports, linguistic quality, and overall report quality. CI = confidence interval; FTR = free-text reports; Max. = maximum rating on the Likert scale; Min. = minimum rating on the Likert scale; SR = structured reports.

	Mean	95%-CI	Min.	Max.	*p*-Value
Trust in reports					
FTR	4.96	4.79–5.13	3	6	<0.001
SR	5.68	5.58–5.78	4	6	
Linguistic quality					
FTR	4.83	4.65–5.01	2	6	<0.001
SR	5.79	5.71–5.87	5	6	
Overall quality					
FTR	5.01	4.86–5.16	3	6	<0.001
SR	5.75	5.66–5.84	5	6	

## Data Availability

Not applicable.
